# The immunopathogenesis of *Helicobacter pylori*-induced gastric cancer: a narrative review

**DOI:** 10.3389/fmicb.2024.1395403

**Published:** 2024-07-05

**Authors:** Maryam Shirani, Saeedeh Shariati, Monireh Bazdar, Faezeh Sojoudi Ghamnak, Melika Moradi, Reyhane Shams Khozani, Elahe Taki, Zahra Arabsorkhi, Mohsen Heidary, Dorsa Bahrami Eskandari

**Affiliations:** ^1^Toxicology Research Center, Medical Basic Sciences Research Institute, Ahvaz Jundishapur University of Medical Sciences, Ahvaz, Iran; ^2^Student Research Committee, Ahvaz Jundishapur University of Medical Sciences, Ahvaz, Iran; ^3^School of Medicine, Razi Hospital, Ilam University of Medical Sciences, Ilam, Iran; ^4^Department of Biology, Rasht Branch, Islamic Azad University, Rasht, Iran; ^5^Department of Microbiology, Faculty of Medicine, Ahvaz Jundishapur University of Medical Sciences, Ahvaz, Iran; ^6^Biotechnology Research Center, Pasteur Institute of Iran, Tehran, Iran; ^7^Department of Microbiology, School of Medicine, Kermanshah University of Medical Sciences, Kermanshah, Iran; ^8^Department of Medical Genetics, National Institute of Genetic Engineering and Biotechnology, Tehran, Iran; ^9^Department of Laboratory Sciences, School of Paramedical Sciences, Sabzevar University of Medical Sciences, Sabzevar, Iran; ^10^Department of Biology, Faculty of Science, Malayer University, Malayer, Iran

**Keywords:** *Helicobacter pylori*, immunopathogenesis, gastric cancer, review, *Helicobacter*

## Abstract

*Helicobacter pylori* infection is a well-established risk factor for the development of gastric cancer (GC). Understanding the immunopathogenesis underlying this association is crucial for developing effective preventive and therapeutic strategies. This narrative review comprehensively explores the immunopathogenesis of *H. pylori*-induced GC by delving into several key aspects, emphasizing the pivotal roles played by *H. pylori* virulence factors, including cytotoxin-associated gene A (*cagA*) and vacuolating cytotoxin A (*vacA*), blood group antigen-binding adhesin (*babA*), and sialic acid binding adhesin (*sabA*). Moreover, the review focuses on the role of toll-like receptors (TLRs) and cytokines in the complex interplay between chronic infection and gastric carcinogenesis. Finally, the study examines the association between *H. pylori* evasion of the innate and adaptive immune response and development of GC. A comprehensive understanding of the immunopathogenesis of *H. pylori*-induced GC is essential for designing targeted interventions to prevent and manage this disease. Further research is warranted to elucidate the intricate immune responses involved and identify potential therapeutic targets to improve patient outcomes.

## 1 Introduction

Malignancy development is a multi-step process, and numerous factors, such as bacteria, viruses, radiation, and chemicals, have been identified to cause cancer (Alipour, 2021). Among all cancers, gastric cancer (GC) is one of the most prevalent types (Rawla and Barsouk, 2019; Guan et al., 2023). Infection with *Helicobacter pylori* (*H. pylori*) is one of the risk factors for this type of cancer (Goh et al., 2014). *H. pylori* is a coccoid or spiral-shaped Gram-negative bacterium that preferentially colonizes the stomach epithelium. This bacterium 3–5 polar flagella that are employed for motility and are oxidase, catalase, and urease positive. The infection by this pathogen is identified as a risk factor for GC, which can vary significantly across different populations with relatively similar rates of *H. pylori* infection (Navashenaq et al., 2021). The World Health Organization (WHO) categorized *H. pylori* as a carcinogen (Alipour, 2021; Reyes, 2023). The eradication of *H. pylori* seems to lower the incidence of GC, according to a 2009 meta-analysis (Fuccio et al., 2009). Almost everyone who has a *H. pylori* infection also has stomach inflammation. However, only a small proportion of colonized people experience clinically obvious consequences. Peptic ulcer, adenocarcinoma, and mucosa-associated lymphoid tissue (MALT) lymphoma are the most common cancers among infected individuals (Ohba and Iijima, 2016). Variations in the expression of bacterial products, changes in the host’s inflammatory responses, and host–microbe interactions may all be associated with increased risk (Israel and Peek, 2001). According to recent research, *H. pylori* infection can result in GC by inducing the disturbance of signaling pathways, abnormal DNA methylation, and gene mutations. Even in healthy mucosa, abnormal DNA methylation and point mutations may accumulate, giving rise to field cancerization (Ohba and Iijima, 2016). In this regard, the effects of gene polymorphisms have been discussed in relation to various interleukins like tumor necrosis factor alpha (TNF-alpha), interleukin-1 β (IL-1β), and IL-10 (Wessler et al., 2017). Three crucial processes make up the very complicated process of *H. pylori* infection: (i) colonization, (ii) immune evasion, and (iii) disease induction (Pop et al., 2022; Ali and AlHussaini, 2024). This pathogen settles in the deep areas of the gelatinous mucous layer lining the gastric mucosa and between the apical surface of epithelial cells and the mucous layer. The bacterium’s capacity to colonize a particular stomach niche is mostly determined by host immune gene polymorphisms and gastric acid secretion (Pop et al., 2022). This colonization of the stomach mucosa is facilitated by bacterial virulence factors such as the vacuolating cytotoxin *VacA* and the cytotoxin-associated gene pathogenicity island-encoded protein *CagA*, which also appear to influence the host’s immune system (Kusters et al., 2006). Herein, this review will focus on the immunopathogenesis of *H. pylori*-induced GC. *H. pylori* infection in the digestive tract can be initiated and sustained by a range of binding antigens and inflammatory proteins on its surface (Leunk et al., 1988; Censini et al., 1996; Tomb et al., 1997; Covacci and Rappuoli, 2000; Lu et al., 2005; Boonyanugomol et al., 2012). This infection triggers inflammatory responses such as the activation of interleukins (Kim et al., 2020; Rashad and Aljanaby, 2021), toll-like receptors (TLRs) (Tran et al., 2024; Zhang et al., 2024), and NF-κB pathways (Lee et al., 2005; Marta et al., 2020), leading to tissue and cellular damage (Zheng et al., 2023). Chronic exposure to these inflammatory processes, coupled with oxidative stress from reactive oxygen and nitrogen species (ROS/RNS) (Han et al., 2022), can weaken the immune defense mechanisms, resulting in tissue injury and increasing the risk of GC.

## 2 Role of *Helicobacter pylori* virulence factors in gastric cancer

There are strong links between GC and *H. pylori*. Several strains of *H. pylori* are reported to facilitate its prolonged survival in the host cell epithelium (Padda et al., 2021). The adhesion factors including blood group antigen-binding adhesin (BabA), sialic acid-binding adhesin (SabA), and outer inflammatory protein A (OipA) attach *H. pylori* to the gastric epithelium (Fagoonee and Pellicano, 2019). Studies have shown that numerous factors play a key role in the progress of the infection and its subsequent progression to GC. Once *H. pylori* has colonized the gastric mucosa, virulence factors, host-relevant factors, and environmental elements, which could increase its impact (Padda et al., 2021). *H. pylori* secrete virulence factors in the form of proteins that enable persistence in the acidic environment of the human stomach, also causing immune evasion. *H. pylori* virulence factors can be classified as three key pathogenic processes: colonization, immune evasion, and disease development. Flagella, urease, and outer membrane proteins are virulence factors involved in colonization, whereas CagA, cagPAI, and VacA are essential for disease induction and host defense (Sukri et al., 2020). The substantial risk of getting GC as a result of *H. pylori* infection could be evaluated by analyzing the research that focuses on these important virulence factors.

### 2.1 Cytotoxin-associated gene

**Figure 1:**
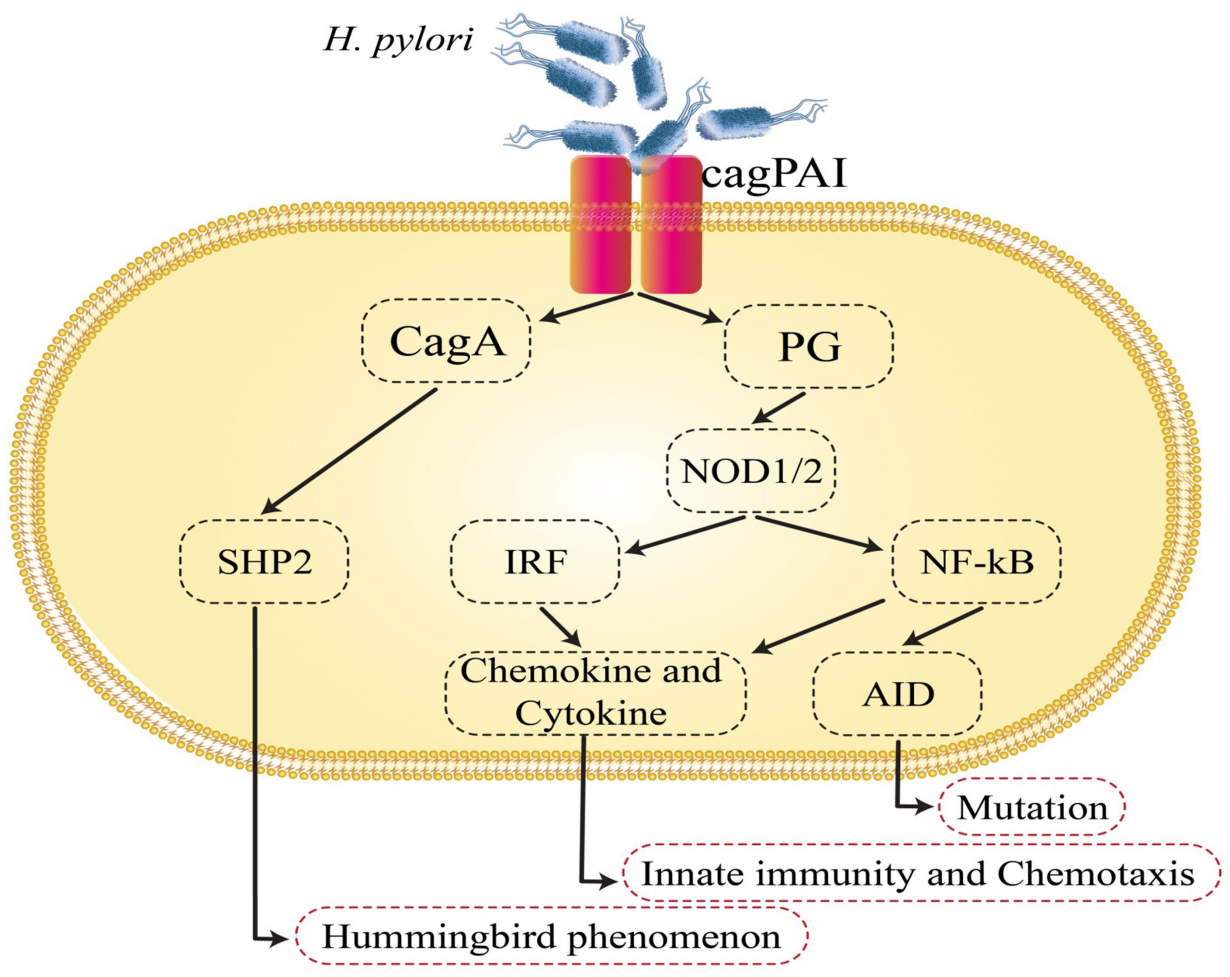
*Helicobacter pylori*-induced intracellular signaling events that are involved in gastric cancer development. AID, activation-induced cytidine deaminase; cagPAI, cag pathogenicity island; NF-κB, nuclear factor-κB; SHP2, Src homology 2-containing protein tyrosine phosphatase.

The cag pathogenicity island (cag PAI) of *H. pylori* represents one of the most potent virulence factors in GC (Sgouras et al., 2015). *CagA* has been found in the early 1990s and has a significant correlation with peptic ulcers and GC. *H. pylori* strains that possess cag PAI have a higher risk of developing gastritis and GC when compared to strains without cag PAI (Tegtmeyer et al., 2011; Haddadi et al., 2020a). *H. pylori* promotes the infection by attaching or adhering to the gastric epithelium of the host. Adhesions and outer membrane proteins (OMP) assist in this process (Tegtmeyer et al., 2011). The cag PAI is a 40 kb DNA insertion element. It includes 27–31 genes, including also the *cagA*, *cagL*, and *cagY* genes, that encode the Cag type IV secretion system (CagT4SS) proteins (Backert et al., 2015). The CagT4SS proteins seem to function as a syringe-like pilus that injects CagA in gastric epithelial cells (Tegtmeyer et al., 2011; Alfarouk et al., 2019), where CagA undergoes tyrosine phosphorylation by Src family kinases or the Abl kinase at the EPIYA motifs found in the C-terminal area (Odenbreit et al., 2000; Higashi et al., 2004; Poppe et al., 2007; Tammer et al., 2007). Thereafter, the supplied CagA binds to and activates Src homology 2-containing protein tyrosine phosphatase (SHP2) (Odenbreit et al., 2000; Higashi et al., 2004), and the CagA-deregulated SHP2 performs diverse roles, including mitogen-activated protein kinase cascade and the activation of the extracellular regulated kinase (Higashi et al., 2004). The regulation of focal adhesion kinase induces the hummingbird phenomenon, which also leads to the elongation and spreading of gastric epithelial cells (Segal et al., 1996; Tsutsumi et al., 2006; Kwok et al., 2007), regulating the inhibition of Src family kinases via stimulating the C-terminal Src kinase, which in turn generates a response regulation circle for the tyrosine phosphorylation cascade, thereby preserving gastric junctional integrity and epithelial polarity (Saadat et al., 2007; Haddadi et al., 2020b). CagA links to Crk, which induces cell scattering. Additionally, it has been demonstrated that CagA activates the nuclear factor of activated T cells by boosting calcineurin, irrespective of its phosphorylation condition (Yokoyama et al., 2005). CagA inhibits cell–cell adherence regardless of CagA tyrosine phosphorylation by breaking tight junctions and inducing a cell’s loss of polarity through suppressing the PAR1/MARK polarity-regulating kinase (Saadat et al., 2007). *H. pylori* acquire access to epidermal growth factor receptors (EGFR) and Her2/Neu by interrupting the tight junctions at the basolateral membrane, which might also enable cell injury and mucosal ulceration (Alzahrani et al., 2014). Additionally, within the cell line of human GC, the CagA–SHP2 complex induces cell elongation by extending the activation of ERK and removing phosphate groups from focal adhesion kinase (FAK). FAK plays a role in the processes of cell adhesion and cell migration (Tsutsumi et al., 2006). CagA has been shown to disrupt the E-cadherin–-catenin complex, leading to β-catenin signal activation (Franco et al., 2005). Each of the following mechanisms of CagA potentially contribute to the progression of GC by creating an environment conducive to the neoplastic transformation of epithelial cells (Figure 1) (Chiba et al., 2008).

### 2.2 Vacuolation cytotoxin A

All *H. pylori* isolates release Vacuolation cytotoxin A (VacA) through the type V secretion pathway, which is the main toxin protein that leads to vacuolation once within the cell (Sheu et al., 2003). VacA is an 88 kDa protein composed of the subunits p33 and p55. The p33 (N-terminal, 33 kDa) section creates an inner chloride transport channel, whereas the p55 (C-terminal, 55 kDa) region is essential for toxin adherence to host cells (Boquet and Ricci, 2012). VacA has multiple biological functions. It attaches to host cells and is internalized, resulting in a severe “vacuolation” defined by a collection of giant vesicles exhibiting characteristics of both late endosomes and early lysosomes. It is suggested that this effect is caused by the introduction of VacA channels that selectively allow anions to pass through cell membranes. These channels promote the influx of chloride ions, elevating the concentration of intraluminal chloride. Consequently, this triggers osmotic swelling and the subsequent formation of vacuoles within the cytoplasm of gastric cells. In addition to its vacuolating impacts, it has recently been proven that VacA directly influences mitochondrial function, impedes T-cell proliferation, and induces apoptosis (Rassow and Meinecke, 2012). Stimulation of dynamin-related protein 1 (DRP1) could play a crucial role in VacA-induced mitochondrial disturbance, as the inhibition of DRP1-dependent mitochondrial fission in VacA-intoxicated cells obstructed Bax activation and mitochondrial outer membrane permeabilization (MOMP) and helped prevent death of intoxicated cells (Jain et al., 2011). VacA also could impair the epithelial cells’ tight junctions and inhibit T lymphocyte proliferation and activation in the lamina propria. Autophagy disruption is an additional method through which VacA promotes gastric inflammation (Palframan et al., 2012; Raju et al., 2012). This disruption permits *H. pylori* to successfully escape the immune system, remain as a chronic infection (Sundrud et al., 2004), and contribute to the development of GC (Palframan et al., 2012; Raju et al., 2012). The existence of *vacA* genotypes strongly predicted the degree of virulence of cagA-positive *H. pylori* strains. According to a South East Asian meta-analysis, the *vacA* s1 allele is commonly detected in *cagA H. pylori* from East Asia and Western Europe and is linked to the improvement of peptic ulcers (Alzahrani et al., 2014).

### 2.3 High-temperature requirement A

*H. pylori* serine protease high-temperature requirement A (HtrA) is an extremely active protein under severe conditions that destroys misfolded proteins in the bacterial periplasm, consequently enhancing bacterial survival in challenging situations (Hoy et al., 2013). Experimental *in vitro* infection demonstrated that the HtrA protein cleaves the extracellular domain of E-cadherin, resulting in the disruption of cell junctions in polarized cell monolayers (Hoy et al., 2010). Multiple investigations have identified the *H. pylori* HtrA protein as an E-cadherin-targeting protease that actively catalyzes the breakdown of the extracellular domain of E-cadherin, thereby breaking cell–cell adhesion and promoting the development of cancer (Hoy et al., 2012; Schmidt et al., 2016a,b; Tegtmeyer et al., 2016).

### 2.4 Blood group antigen-binding adhesin

Blood group antigen-binding adhesin (BabA) is a protein located in the outer membrane of *H. pylori*. It specifically recognizes Lewis b blood group antigens (Leb) present on the gastric epithelial cells of the host. The presence of BabA in *H. pylori* strains appears to contribute to increased virulence and a higher likelihood of successful bacterial colonization (Chang et al., 2018). While three alleles of Bab (BabA1, BabA2, and BabB) have been identified, attachment to Leb requires just the BabA2 genotype (Roesler et al., 2014). BabA binds to host epithelial cells to facilitate the translocation of CagA through into host cell by T4SS (Kable et al., 2017). The expression of CagA, VacA, and BabA2 increases the degree of *H. pylori* infection because these virulence factors operate synchronously. This finding will result in a more intense inflammatory response and an increased risk for GC (Roesler et al., 2014). According to a meta-analysis of case–control studies, babA2 genopositive status is associated with an increased risk of PUD (OR = 2.07) in Western nations but not in Asian ones (Chen et al., 2013).

### 2.5 Sialic acid-binding adhesin

The significance of sialic acid binding adhesin (SabA), a new outer membrane protein, in the pathophysiology of gastroduodenal disease is becoming more apparent. During the initial phase of infection, the binding of blood group antigen-binding adhesin (BabA) to Lewis b antigen and related fucosylated ABO blood group antigens is likely crucial. Throughout an inflammatory response by the host, there is increased expression of Sialyl-Lewis X, which, in conjunction with SabA, enables a greater adherence of *H. pylori* to the gastric mucosa (Yoshio, 2008). It has been shown that SabA production is linked to severe intestinal metaplasia, gastric atrophy, and the onset of GC in both industrialized and developing nations; this finding highlights the need for further study of SabA in the latter (Yoshio, 2008). Furthermore, SabA can mimic selectin to activate the neutrophils. This activation leads to the production of reactive oxygen species (ROS), which further enhances the prolongation of the inflammatory response (Unemo et al., 2005). Multiple roles for gamma-glutamyl transpeptidase (GGT) have been established. *H. pylori* colonization of the gastric mucosa is facilitated, and apoptosis of gastric epithelial cells is induced. Additionally, *H. pylori* GGT suppress T-cell-mediated immunity and dendritic cell growth and differentiation, hence inducing immunological tolerance. *H. pylori* carries GGT in its outer membrane vesicles. Increased levels of H_2_O_2_ and Il-8 synthesis in gastric epithelial cells have been linked to this condition (Gong et al., 2010).

### 2.6 Outer inflammatory protein A

The outer membrane proteins of *H. pylori* [HomB, HopQ, and HopH (OipA)] are linked to the development of GC (Cover, 2016; Braga et al., 2019). No receptor for outer inflammatory protein antigen (OipA) has been discovered (Posselt et al., 2013). OipA, which is encoded by the *HopH* gene, is an outer membrane protein associated with inflammation. The inflammatory response caused by OipA positive *H. pylori* strains is greater than that caused by OipA negative strains. This finding raises the probability of developing GC and gastric ulcer disease (Zhang et al., 2014). Patients with precancerous gastric lesions are more likely than individuals with simple gastritis to have OipA in their gastric biopsies (Baj et al., 2021). Furthermore, the Bcl-2 pathway is involved in the apoptotic cascade activation upon OipA binding to gastric cells (Teymournejad et al., 2017). Several proinflammatory cytokines, including IL-6, IL-8, and, IL-1, are believed to be stimulated by OipA. Increased GC risk is a result of its effect on IL-10 secretion and dendritic cell maturation (Teymournejad et al., 2014). The OipA “on,” but not “off,” state has also been observed to be strongly associated with an increased risk of PUD (OR = 3.97) and GC (OR = 2.43), particularly in Western nations, according to a meta-analysis (Liu et al., 2013).

### 2.7 Duodenal ulcer promoting gene

In the adaptability zone of *H. pylori,* a new gene that is highly homologous to the *virB4* gene, named duodenal ulcer stimulating gene A (*dupA*), has been discovered. Recent evidence indicates that *dupA* is part of a novel cluster of vir homolog genes expected to generate a new T4SS that contributes to the emergence of *H. pylori*-related pathologies (Yamaoka, 2008; Fischer et al., 2010). Lu et al. found that infection with *dupA*-positive strains was substantially linked with duodenal ulcers but inversely correlated with GC after screening 500 *H. pylori* strains collected from patients in Colombia, Japan, and South Korea (Lu et al., 2005). Even though other studies have not clarified those same research results with *dupA*, they are particularly appealing considering that other recommended *H. pylori* virulence genes have also been linked to both GC and ulcer disease, while patients with duodenal ulcers contradictory have a lower risk of developing GC (Hansson et al., 1996). Table 1 summarizes all virulence factors and their role in GC.

**Table 1:** Virulence factors and their role in GC.

Virulence factor	Association with GC	Role
Cytotoxin-Associated Gene (cagA)	Strongly associated with gastritis and gastric adenocarcinoma	Inducing cell elongation, disrupting cell junctions, and activating signaling pathways associated with cell proliferation and migration
Vacuolation Cytotoxin A (VacA)	Associated with gastritis and gastrointestinal tract adenocarcinomas	Released by all *H. pylori* isolates via the type V secretion pathway, this induces vacuolation in host cells by forming chloride transport channels that lead to osmotic swelling and subsequent vacuole formation while also impacting mitochondrial function, impairing T-cell proliferation, and disrupting tight junctions
High-Temperature Requirement A (HtrA)	Associated with GC	Heightened activity under adverse conditions, aiding bacterial survival, and notably, experimental evidence reveals its role in disrupting cell junctions by cleaving the extracellular domain of E-cadherin
Blood Group Antigen-Binding Adhesin (BabA)	Associated with GC	Binds to host blood group antigens, facilitating adherence to gastric epithelium and synchronously operating with other virulence factors like VacA and CagA, thereby intensifying inflammatory responses
Sialic Acid-Binding Adhesin (SabA)	Associated with GC	Promoting bacterial adherence to the gastric mucosa, contributing to severe gastric conditions such as intestinal metaplasia and gastric atrophy, and triggering inflammatory responses
Outer Inflammatory Protein A (OipA)	Associated with GC	Eliciting a robust inflammatory response, enhancing the probability of gastric cancer and ulcer disease progression, and modulating apoptotic pathways and cytokine secretion, thereby elevating the risk of gastric cancer and peptic ulcer disease
Duodenal ulcer promoting gene (dupA)	Not specifically associated with GC	Controversial; some studies suggest a link to duodenal ulcers., while its mechanism of action is suggested to involve its participation in a novel cluster of vir homolog genes, potentially leading to the formation of a new type IV secretion system (T4SS) that influences the pathogenicity of *H. pylori* strains

## 3 Role of toll-like receptors in *Helicobacter pylori*-induced gastric cancer

**Figure 2:**
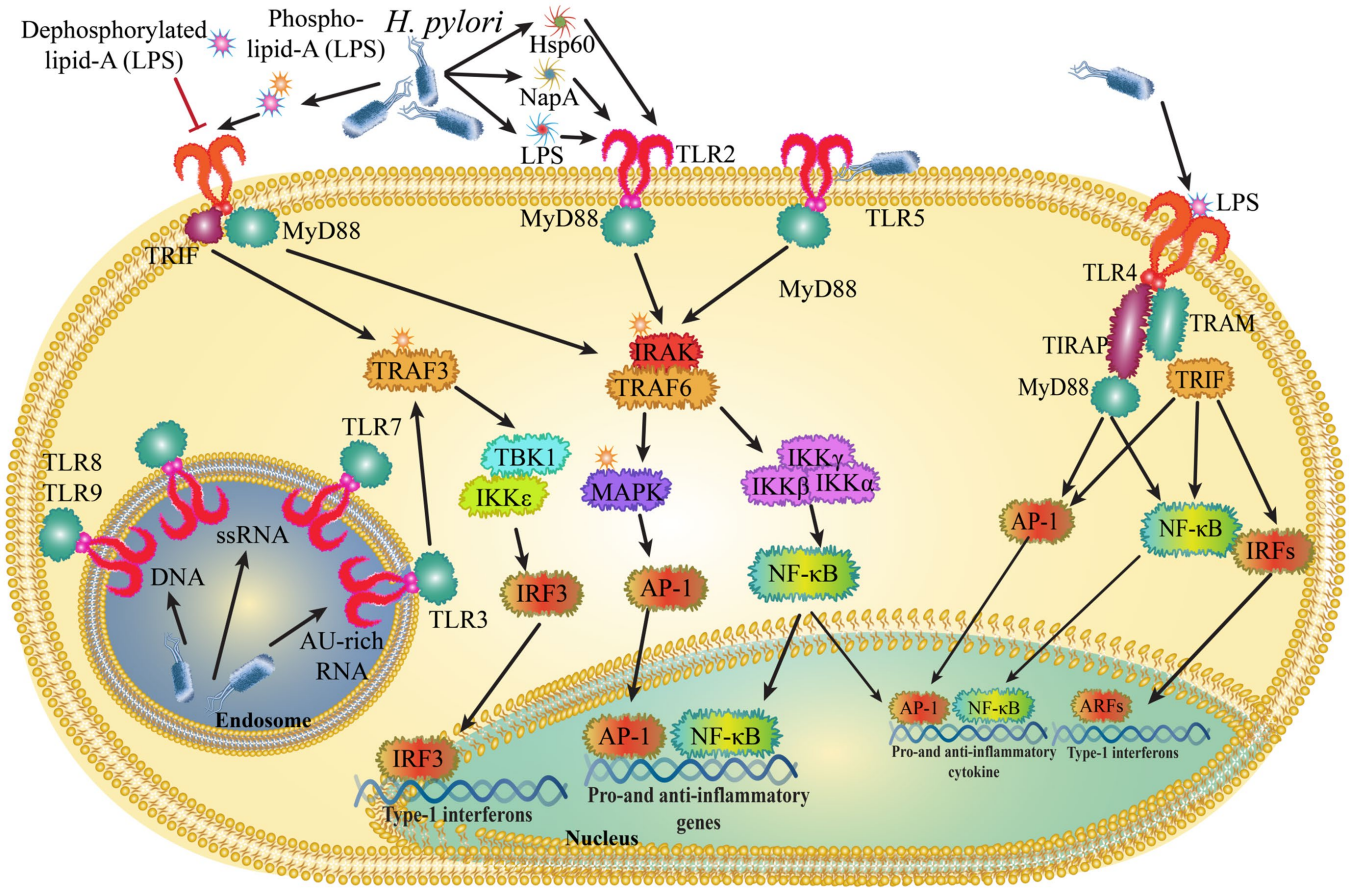
*Helicobacter pylori* control innate immune signals by interacting with toll-like receptors (TLRs).

Toll-like receptors (TLRs) play a key role in the signaling of numerous pathogen-related compounds and endogenous proteins linked to immune activation. The initial line of innate immunological protection against *H. pylori* is provided by the stomach mucosa’s gastric epithelial cells, which respond to infections by starting a variety of cell-signaling cascades. Numerous of these cell signaling events have been revealed to be mediated by pathogen recognition receptors (PRRs) of the TLR family (Smith, 2014). The generation of pro-inflammatory cytokines, chemokines, and reactive oxygen species by the TLR pathways activated by *H. pylori*-induced inflammation has been demonstrated to be strongly linked not only to gastric carcinogenesis but also to the development of the tumor microenvironment (Figure 2).

### 3.1 Role of TLR1

A link between a variant of the TLR1 genetic locus (4p14) and *H. pylori* seroprevalence was found in a meta-analysis conducted in Europe. The observed difference in each person’s susceptibility for *H. pylori* infection may be partially explained, if verified, by genetic polymorphisms in TLR1. This finding may also support the idea that *H. pylori*-related carcinogenesis and genetic polymorphisms of TLRs are related (Mayerle et al., 2013). However, research has identified single-nucleotide polymorphisms (SNPs) of TLRs and their associations with the emergence of GC. Lipoarabinomannan (LAM), peptidoglycans, and bacterial lipoproteins all function as signal-transducing receptors through TLR2.

### 3.2 Role of TLR2

TLR2 was crucial for the release of the pro-inflammatory cytokines IL-1 and IL-6 as well (Obonyo et al., 2007). The promoter activity of TLR2 may be impacted by a polymorphism in TLR2 between positions 196 and 174 caused by a 22-bp deletion. TLR2-196 to -174del polymorphism and the onset of GC were found to be positively correlated in studies from Brazil and Japan, but studies from China and Japan found the link to be negatively correlated (Hishida et al., 2010; Zeng et al., 2011). The results of the other Chinese study showed that neither *H. pylori* infection nor poor prognoses are linked to TLR2 polymorphism and that the del/del genotype is related to an elevated risk of GC in the southern Chinese population (Huang et al., 2019). The risk of all malignancies increased from −196 to −174del, according to a meta-analysis by Gao et al. (2019).

### 3.3 Role of TLR4

TLR4 is normally found in the gastric mucosa and epithelial cells in two distinct parts: the apical and basolateral compartments. In GC tissues, TLR4 expression is increased, leading to enhanced responsiveness to lipopolysaccharide (LPS) from *H. pylori*. This increased response activates the NF-κB pathway and promotes the activation of the IL-8 promoter. TLR4 is highly polarized and expressed in the apical and basolateral compartments of normal gastric mucosa and gastric epithelial cells. In GC tissues, TLR4 expression is increased, and this confers LPS responsiveness, which enhances the activation of the NF- κB and IL-8 promoters in response to stimulation with *H. pylori* LPS. When LPS binds to TLR4 in gastric epithelial cells, TLR4 signaling is activated, which leads to the production of inflammatory mediators including IL-8 and TNF-α, which are essential for the progression of tumor. Studies have revealed that TLR4 is overexpressed in gastric epithelia and that it is also elevated in the monocytes and macrophages of superficial gastritis in cases of GC (Figure 2) (Ito et al., 2020). According to a new study, *H. pylori* attaches to human annexins using LPS in order to prevent TLR4 signaling (Schmidinger et al., 2022). TLR4 polymorphism (Asp299Gly) can reduce responsiveness to *H. pylori*-LPS by lowering the affinity of bacterial ligands for binding. These modifications may contribute to the development of long-lasting infections, following chronic inflammation, and cancer. There are contradictions in the relationship between the incidence of GC and TLR4 polymorphism. According to the findings of a Japanese investigation, the TLR4 + 3,725 G/C polymorphism was a risk factor for severe gastric atrophy in Japanese people who were seropositive for *H. pylori*. The relevance of the differences in host innate immunity caused by TLR4 polymorphism as genetic predispositions to gastric precancerous lesions in Eastern Asian groups with similar backgrounds was highlighted by these results (Hishida et al., 2009). Another Japanese investigation found that having the TLR4 + 3,725 C allele and the miR-146a rs2910164 G/G variant together improved the likelihood of severe gastric atrophy in *H. pylori*-infected Japanese participants (Hishida et al., 2011). The correlation between the TLR4 Asp299Gly and TLR2-196 to -174del polymorphism and the likelihood of developing GC was also supported by a study from China (Castaño-Rodríguez et al., 2013). TRL4 gene variants, however, were not connected to gastric carcinogenesis in Caucasians, indicating that this gene variant should not be used as a potential biomarker to identify people at higher risk of developing GC (Kupcinskas et al., 2011).

### 3.4 Role of TLR5

Despite the fact that *H. pylori* flagellin has been proposed as a TLR5 ligand, its activity as a TLR5 activator is modest, indicating a potential mechanism that contributes to *H. pylori* persistence (Lee et al., 2003; Smith et al., 2003; Gewirtz et al., 2004). Through an ERK-dependent mechanism, flagellin-activated TLR5 increases the proliferation of GC cells (Song et al., 2011). Due to its tendency to interfere with ligand recognition, the TLR5rs5744174 polymorphism has also been linked to an increased risk of gastric carcinogenesis in Chinese patients, allowing *H. pylori* to survive in the stomach (Zeng et al., 2011). Two more TLR5 polymorphisms (rs1640827 and rs17163737), according to a recent Chinese study, were linked to *H. pylori* infection and a high risk of GC (Xu et al., 2017). In intracellular vesicles such the endosome, lysosome, and endoplasmic reticulum, TLR3, TLR7, TLR8, and TLR9 are present. They play a major role in the identification of microbial nucleic acids (Kawai and Akira, 2009; Kawai and Akira, 2011).

### 3.5 Role of TLR8, TLR9, and TLR10

TLR8 on dendritic cells, which is endosomally localized, may detect the RNA of *H. pylori*. TLR9 can be activated by an inflammatory milieu that comprises cells without polarity, promoting proinflammatory cascades that ultimately lead to the development of GC (Schmaußer et al., 2004; Wang et al., 2014). One study found that *H. pylori*’s specific TLR-9 activation is dependent on the cag T4SS but not on virD4 coupling proteins or virD2 relaxases (Tegtmeyer et al., 2022). As the only receptor with dual pro-and anti-inflammatory functions, TLR9 plays a dichotomous role. The microenvironment, particularly the presence of *H. pylori*, affects its function (Meliț et al., 2019). A functional receptor called TLR10 participates in the innate immune reaction to *H. pylori* infection (Nagashima et al., 2015). The TLR10 rs10004195 polymorphism may be important for gastric pathophysiology and *H. pylori* susceptibility (Tongtawee et al., 2018). Discovering diagnostic/predictive biomarkers and therapeutic targets for GC needs to be the subject of further studies on the regulation of TLRs in *H. pylori*-associated gastric carcinogenesis.

## 4 Role of cytokines in *Helicobacter pylori*-induced gastric cancer

### 4.1 Role of SOCS1 hypermethylation and mediators of the JAK/STAT pathway

According to Iqra Jan et al., *H. pylori* infection enhances IL-6, IL-10, and TGF-b in the physical excursion, even though the critical function of the cytokine to signal in the induction of epigenetic corrections within GC is yet unclear. Although IL-1 and IL-6 help overactivate the JAK/STAT passage, *H. pylori* infection causes the disablement of the inhibitor of cytokine signaling one (CS1) gene between the promoter’s amazing methylation and the promoter’s area. Furthermore, *H. pylori*-mediated epigenetic turn of CS1 within harmony by the over-activation of the inflammatory cytokines group of the JAK/STAT passage on GC (Sheh et al., 2010). Predictive models for virulence gene play a significant role in clinical consequences. In other words, cytotoxin-associated gene A (*cagA*) and phosphor glucosamine mutase gene (*glmM*) are the critical genes in the *H. pylori* pathogenicity pathway (Torres et al., 1998; Beevers et al., 2004; Šebunova et al., 2018).

Several immune cells including macrophages, monocytes, mast cells, neutrophils, Th17, Th2, and Th1 produce the cytokines in *H. pylori*-induced GC. For example, TNF-a, TGF-b, IFN-g, CXCL12, CXCL4, IL-10, IL-17, IL-23, IL-1, IL-2, and IL-6 cytokines are secreted in widespread blood circulation (Vorobjova et al., 1998; Niu et al., 2020). Gastric epithelial cells have many specific membrane receptors that pass on the signal conveyed through various cytokines inside the cell (Zhuang et al., 2012). The cytokine’s role in different types of cells is their attachment to related membrane receivers and their adjustment of downstream factors to signal cascade (Bockerstett and DiPaolo, 2017). Multiplex cytokines stimulate Janus kinase 2 (JAK2) and lead to the signal phosphorylate converter. Furthermore, these cytokine activators of transcription 3 (STAT3) such as phosphorylated STAT3 enter the nucleic acid regulatory sequences and leads to GC development (Pandey et al., 2018). The suppressor of cytokine signaling-1 (SOCS1), reacting to phosphoserine, acts within the JAK kinase pathway to regulate the stimulation of STAT proteins or another signaling transitional intermediates in the JAK/STAT signaling cascade. Furthermore, the SOCS1 has been suggested to be in charge of GC. IL6 and TFGb are essential for controlling the expression of SOCS1.

### 4.2 Role of interleukin-32

According to Kosuke Sakitani et al., the real-time RT-PCR results *in vitro* show that *H. pylori* increase protein expression in infectious stomach cells. Next, in a time-dependent procedure, the Western blotting analysis showed that *H. pylori* induced interleukin-32 in the GC cell lines (Censini et al., 1996). IL-1 and TNF-α have been introduced to highly induce IL-32 in a few cell lines (Bockerstett and DiPaolo, 2017).

### 4.3 Cytokines produced by epithelial cells

According to research, cytokines produced by epithelial cells, particularly IL-33, play a role in the development of GC. IL-33 is expressed in gastric epithelium, specifically in surface mucous cells. It binds to ST2 (IL1RL1) receptor and activates the NF-kB and MAPK signaling pathways through its subsidiary protein IL-1RAP. In a study by Bockerstett et al., inflammation, atrophy, and metaplasia in the fundus of the stomach were induced via an IL-13-dependent mechanism. IL-6 and IL-11, members of the IL-6 family of cytokines, along with gp130 signaling, were found to be connected to JAK–STAT signaling pathways and contributed to atrophic gastritis. Additionally, cytokines from the IL-12 family, particularly IL-23, were observed to be altered in GC patients. It should be noted that gp130 signaling is frequently disrupted in GC. These discoveries have highlighted the significance of IL-33, IL-11, and other cytokines in GC (Bockerstett and DiPaolo, 2017).

### 4.4 Inflammation induced by *Helicobacter pylori*

The mechanism of *H. pylori* for the induction of pro-inflammatory cytokines is presumed to be the outer membrane adhesion proteins such as OipA and BabA, which induced IL-6 to IL-11 production via elimination; however, the mechanism is unclear (Sugimoto et al., 2011). One of the immunosuppression T-cell stimulation permits the durability of the bacterial infection. Finally, NF-B stimulates by targeting T-cells (Takeshima et al., 2009). The CagA2 duty offers a position in NF-B and IL-8 activation (Backert and Naumann, 2010). Although CagA does not show to be the leading player in *H. pylori*-induced IL-12 generation, it exclusively seems to be a *H. pylori* strain (Viala et al., 2004). Furthermore, stimulating cytokine rescue, *H. pylori* motivates the generation of growth elements, for example, granulocyte-macrophage colony-stimulating factor (GM-CSF) and oxygen/nitrogen species (ROS/RNS) cyclooxygenase-2 (COX-2). *H. pylori*-induced GC and persuadable nitric oxide synthase (iNOS) generate prostaglandin E2 and nitric oxide (Cho et al., 2010). LPS of *H. pylori* plays a prominent role in transforming iNOS, leading to an increased NO release (Cavallo et al., 2011). No synthesis can be regulated between host arginase and *H. pylori* arginases II (Gobert et al., 2011). In culture, *H. pylori* sound waves influenced oxidative bursts from neutrophils and monocytes (Hansen et al., 1999; Marta et al., 2020). HP-NAP induces ROS release from neutrophils and promotes recruitment of other leukocytes to the site of infection by stimulating the production of chemokines such as CXCL8, CCL3, and CCL4 (Dundon et al., 2002).

### 4.5 Activation of NF-κB, the primary regulator of inflammation

**Figure 3:**
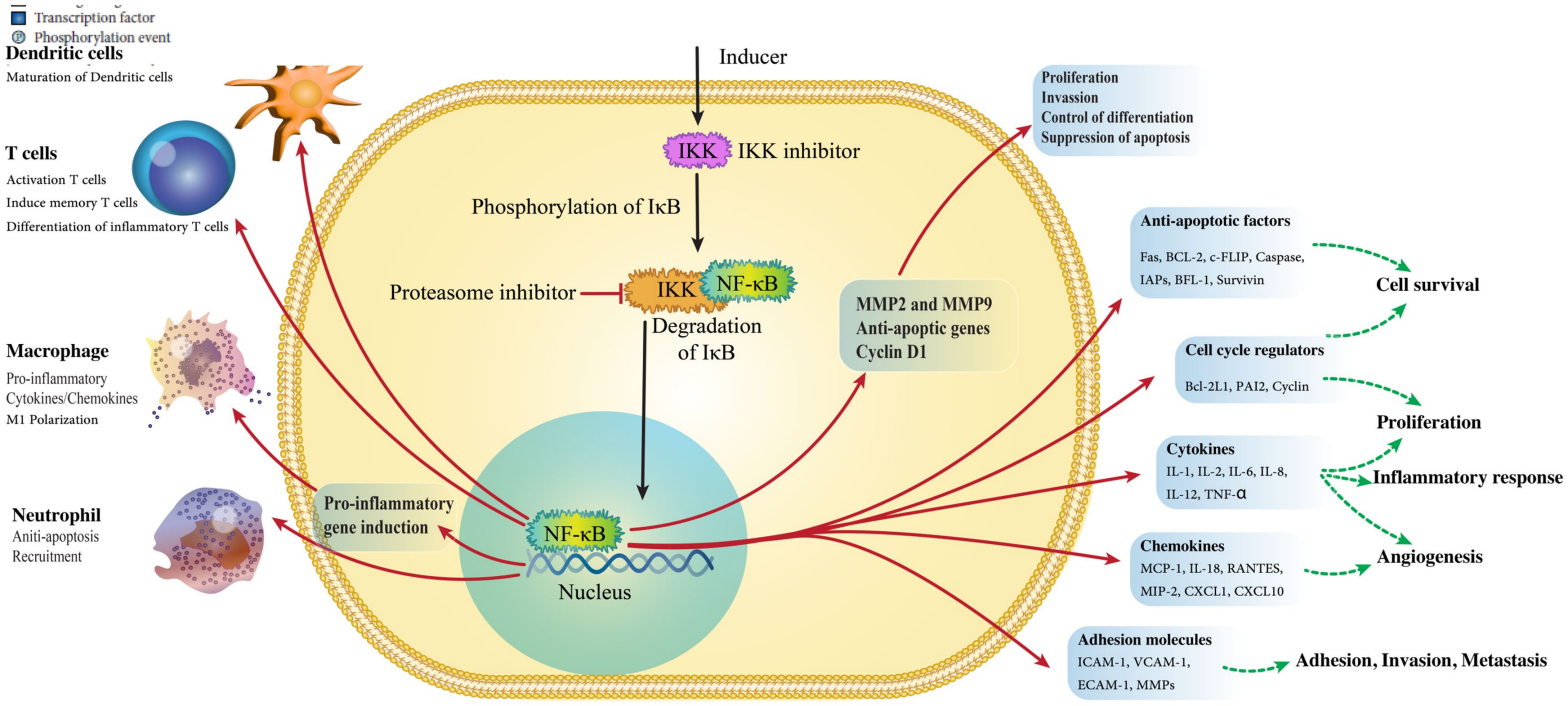
Activation of NF-κB, the primary regulator of inflammation.

NF-κB is the primary regulator in the inflammatory response and adjusts many cellular proceedings necessary in *H. pylori*-induced GC (Figure 3) (Orlowski and Baldwin, 2002). Various pro-inflammatory triggers can activate NF-κB, including TLR activation with pathogens generated by cytokines through both common and non-common pathways (Hayden and Ghosh, 2008). In the common pathway, the different ligands bind to receptors causing the stimulation of the IB kinase (IKK), which contains IKK, and NEMO/IKK. This kinase mixes phosphorylates and destroys the suppressor of IB, ultimately leading to cell growth (Chen and Greene, 2004).

In the uncommon pathway, this signal from receptors causes the activation of NF-κB inducing kinase (NK) and IKK, which, in turn, phosphorylates p100 and converts it into p52. Then *H. pylori* infection activates NF-κB in both pathways (Lamb and Chen, 2010; Sun, 2011). Through the common pathway, NF-κB is activated. However, in immune cells, such as B-lymphocytes, *H. pylori* infection stimulates NF-κB, and the connector was challenging to remove and appears too plentiful. The host cells banded to TLR2 or TLR4 are recognized by *H. pylori* LPS, and then *H. pylori* induces NF-κB stimulation and inflammatory response (Evans et al., 1995). According to Mandell et al. (2004), not only was *H. pylori* LPS a very weak activator of TLRs but it also aids the bacteria’s sustainability and has a low-degree inflammation through the stomach (Mandell et al., 2004). Despite this finding, *H. pylori* LPS could be significant in activating inflammatory pathways in leukocytes, responding to bacterial colonization. According to previous investigations, macrophages and monocytes illustrate the value of TLRs in gastric epithelial cells (Maeda et al., 2001; Obonyo et al., 2007). Peptidoglycan in *H. pylori* was an effective molecule identified by stomach epithelial cells and nucleotide oligomerization domain 1(NOD1) (Grubman et al., 2010). Then, NOD1 signaling activates MAPKs. In conclusion, inflammatory cytokines, such as IL-32 and IL-8, can be useful for drug treatment. The investigation of gene-targeted mice showed that Th1 cytokines drawback GC induced by *H. pylori* (Smythies et al., 2000). The *H. pylori* -induced GC discharged peptidyl-prolyl cis (Kumar et al., 2021). Table 2 summarizes all host factors and their role in GC.

**Table 2:** Summary of the host factors that play a significant role in the development and progression of gastric cancer related to *H. pylori* infection.

Host factor		Function	References
Toll-like receptors (TLR)		Generation of pro-inflammatory cytokines, chemokines, and reactive oxygen species activated by *H. pylori*-induced inflammation that is linked to gastric carcinogenesis	Mayerle et al. (2013)
TLR	TLR2	Stimulation of the pro-inflammatory cytokines IL-1 and IL-6 (The relationship between polymorphism in TLR2 and onset of GC)	Obonyo et al. (2007), Zeng et al. (2011), Hishida et al. (2010)
	TLR4	Activation of signaling, which leads to the production of inflammatory mediators (IL-8 and TNF- α) that are essential for the progression of tumor (The relationship between polymorphism in TLR4 and onset of GC)	Ito et al. (2020), Schmidinger et al. (2022), Hishida et al. (2009)
	TLR5	Increasing the proliferation of GC cells through an ERK-dependent mechanism (The relationship between polymorphism in TLR5 and onset of GC)	Song et al. (2011), Xu et al. (2017)
	TLR8,9,10	Promoting pro-inflammatory cascades that ultimately lead to the development of GC (The relationship between polymorphism in TLR10 and onset of GC)	Schmaußer et al. (2004), Tongtawee et al. (2018)
IL-1, IL-6, and IL-11		Over-activation of the JAK/STAT pathway leading to GC	Sheh et al. (2010)
Suppressor of cytokine signaling-1 (SOCS1)		Activation of JAK/STAT signaling cascade and hyperactive methylation of SOCS1 is related to different GC cell lines	Natatsuka et al. (2015), To et al. (2004)
IL-32		Induction of the expression of pro-tumorigenic factors like IL-8 (overexpression of IL-32 is correlated with the presence of *H. pylori* infection and the development of GC)	Tegtmeyer et al. (2011)
IL-33		Binding to ST2 (IL1RL1) receptor and activates NF-kB and MAPK signaling pathways	Bockerstett and DiPaolo (2017)
NF-κB		Upregulation of various pro-tumorigenic factors, including inflammatory cytokines (IL-1, IL-8), and matrix metalloproteinases (MMP-2, MMP-9) that facilitate tumor invasion and metastasis	Orlowski and Baldwin (2002)

GC, Gastric cancer; TLR, Toll-like receptors; IL, interleukin; TNF, Tumor Necrosis Factor; JAK/STAT pathway, Janus kinase/signal transducers and activators of transcription; ERK, extracellular-signal-regulated kinases; NF-kB, Nuclear factor kappa-light-chain-enhancer of activated B cells; MMP, matrix metalloproteinases.

## 5 Association between *Helicobacter pylori* evasion of immune response with gastric cancer

### 5.1 Colonization and penetration of *Helicobacter pylori* in basic lumen

Bacterial colonization is not possible in the stomach lumen because of its acidic pH. As a result, the lower bowl, which has a normal or slightly alkaline pH, has the largest bacterial population. The production of gastric acid in the stomach results in a pH of 1–2, which inhibits bacterial colonization in this area (Sr et al., 2005; Schreiber et al., 2006). To prevent bacterial infection, the mucous membrane layer of the stomach works as a physical barrier against bacterial invasion and accumulates host antimicrobials. In gastric lumen, mucins transform to a gel state at low pH (Rawla and Barsouk, 2019; Alipour, 2021), which traps bacteria (Celli et al., 2009) *H. pylori* produces urease to alter acidity and reduce mucus concentration by ammonium ions. Therefore, suitable conditions are provided for the entry of *H. pylori* (Sidebotham et al., 2003). These situations emphasize the importance of *H. pylori* in managing its interaction with lumen gastric lumen epithelium until it is able to evade the host immune system defense and stay alive in this condition (Suarez et al., 2006).

### 5.2 *Helicobacter pylori* cytotoxin associated gene

The initial attachment of *H. pylori* to surface receptors on the host gastric epithelial cell is the most crucial event in the development of cancer. Cag A and Vac A, the two important protein toxins that are expressed by pathogenicity island (PAI), are essential for bacterial maintenance and entrance through gastric lumen (Nejati et al., 2018). Cag A is phosphorylated after entering the cell, causing cell proliferation as well as the destruction of strong connections between the adjacent cells (Talebkhan et al., 2008). The presence of Cag A increases the risk of peptic ulcers or GC by 50–70% (Al-Ghoul et al., 2004). Cag A-positive bacteria strains have been highly linked to an increased risk of GC in recent years (Ghotaslou et al., 2018). VacA is actually a toxin that causes cavities in cell membranes that cause vacuoles to form in the cell, and *H. pylori* damages the host cell with the help of this toxin. All strains of *H. pylori* have the *vacA* gene, of which only 50–60% show cytotoxic activity (Soleimani et al., 2016). This factor stimulates vacuole production and ultimately causes cell death through apoptosis (Nejati et al., 2018). VacA also increases the permeability of epithelial cells, which provides nutrients for bacterial growth. This toxin causes erosion of the epithelial cells (Wang et al., 2008).

### 5.3 Escaping *Helicobacter pylori* in the innate immune system

Given that many pathogens cause the survival of *H. pylori*, the main factor is that *H. pylori* impair innate immune responses. Important barriers that can be said to escape *H. pylori* from the immune system include mucus secreted by epithelial cells and innate immune cells (Chmiela et al., 2017). The recognition of conserved pathogen-related molecular patterns (PAMPs) by pattern recognition receptors on epithelial and innate immunity cells in lamina propria of gastric lumen begins with immunological responses against *H. pylori*, which are subsequently followed by adaptive immune responses (Fukata and Abreu, 2009; Mogensen, 2009). Innate immunity in eukaryotes is known as the first line of defense against infections. The main categories of PPRs that identify PAMPs are TLRs-like receptors. Bacterial LPS, peptidoglycans, lipoproteins, lipoic acid, and CpG-rich regions not methylated from DNA are the main targets of TLRs (Takeda and Akira, 2004). Adaptor proteins are activated by TLRs, which then activate nuclear factor NF-κB, interferon regulatory factor (IRF), and activator protein-1 (AP-1). The release of inflammatory cytokines and chemokines, as well as INF-α and INF-β, is triggered when these transcription factors are activated (Kawasaki and Kawai, 2014). TLRs are unable to recognize *H. pylori* due to a variety of PRRs that are required to identify other Gram-negative gut infections (Peek et al., 2010).

#### 5.3.1 Complement evasion

The primary agents of innate immunity include macrophages, neutrophils, dendritic cells, and natural killer cells. Essential processes within innate immunity encompass phagocytosis, the release of inflammatory molecules, triggering of complement system proteins, and the production of acute phase proteins, cytokines, and chemokines (Medzhitov and Janeway, 2000; Ali et al., 2019). Complement is a crucial component of the innate immune system, serving as the primary line of defense against the invading bacteria. The complement response occurs specifically on the bacterial surface, with the breakdown products either binding to the surface or being released into the surrounding fluid. These released chemotactic peptides attract phagocytes from the bloodstream to the infection site, while the abundant C3 fragments aid in bacterial recognition by phagocytes and promote phagocytosis. Ultimately, the formation of the pore-forming membrane attack complex (MAC) directly leads to bacterial eradication by disrupting the bacterial membrane (Berends et al., 2014). To prevent harm to the host, the complement system is meticulously controlled by various proteins present in bodily fluids and on cell surfaces. CD59, also called protectin, is a human regulator of the complement system anchored to the cell membrane through a glycophosphoinsitol (GPI) linkage. This protein shields host cells from destruction by the MAC. Bacteria have been observed to exploit these complement regulators to evade MAC-induced destruction (Blom et al., 2009). For instance, *H. pylori* have been identified to sequester the soluble form of CD59, which is often released from host cell membranes, incorporating it into their outer membrane (OM) to thwart MAC-mediated killing (Rautemaa et al., 1998). Vitronectin (Vn), present in the extracellular matrix and blood plasma, plays a crucial role in various biological functions, such as regulating the complement system. In addition to its involvement in coagulation regulation, Vn, a prominent serum protein, can interact with fluid-phase C5b67 complexes to inhibit the formation of an active MAC. *H. pylori* utilizes vitronectin to evade MAC-induced destruction (Ringnér et al., 1992). *H. pylori* employs a strategy known as moonlighting wherein a single protein serves multiple, unrelated roles to present the hydrogen peroxide-neutralizing enzyme catalase (KatA) on its surface. By binding to Vn, the bacteria can evade the complement system (Richter et al., 2016).

### 5.4 *Helicobacter pylori* evading the adaptive immune system

In the immunological response to *H. pylori*, CD 4+ T cells are more important than CD8 T cells as adaptive immune factor cells*. H. pylori* infection has been associated with CD4 + T cell subsets such as Th1, Th17 and (regulatory T cells) Tregs. Th1 cell activation causes the production of interferon alpha and gamma; Th7 cells also activate interleukins 17, 22, and 21 (Bagheri et al., 2018). The neutrophil-activated protein *H. pylori* (HP-NAP) causes neutrophils and monocytes to generate IL-12, which boosts Th1 responses and increases the production of interferon-gamma (Amedei et al., 2014). Th17 cells appear to be critical for *H. pylori* clearance by attracting neutrophils (Luzza et al., 2000). One of the causes of *H. pylori* infection is that the responses of the effective T cells are mainly disrupted during the infection, leading to a low response of the T cells. T cell proliferation is inhibited by VacA in two ways; first, Vac A interacts with an unknown receptor on T cells, which inhibits cell growth by rearranging actin. Second, Vac A attaches to mitochondria and cause apoptosis and inhibits T-cell growth through this route (Abadi, 2017). The extraordinary induction of Tregs by microbial antigens could be a way for *H. pylori* to evade the immune system (Oertli et al., 2013). Tregs, which are defined by the expression of transcription factor FOXP3, CD25, and the production of IL-10, may modulate the gastric mucosal inflammatory response to *H. pylori*. Other T cell’s cytokine production and proliferation can be suppressed by Tregs (Algood and Cover, 2006).

## 6 Conclusion

This narrative review has provided a comprehensive overview of the immunopathogenesis of *H. pylori*-induced GC, shedding light on the intricate interplay between the bacteria and the host immune system. We have explored several key themes in this study, starting with the central role of *H. pylori* virulence factors in driving carcinogenesis. It is notable that virulence factors such as CagA and VacA act as drivers of carcinogenesis by disrupting host cell functions, fostering chronic inflammation, and promoting malignant transformation. Furthermore, the review examined the pivotal involvement of TLRs and how *H. pylori* cleverly evades the host immune surveillance by altering TLR signaling, thereby sustaining chronic inflammation within the gastric mucosa. This persistent inflammation, in turn, primes the stage for the development of GC, emphasizing the importance of understanding the interplay between bacterial evasion strategies and the host’s innate immune defenses. We have also explored the critical role of cytokines in the context of *H. pylori* infection and GC, elucidating how the dynamic interplay between pro-inflammatory and anti-inflammatory cytokines shapes the microenvironment, ultimately influencing the risk of GC development. Finally, our discussion has highlighted the undeniable association between *H. pylori*’s cunning evasion tactics and the predisposition to GC. These evasion strategies contribute to immune system dysfunction, chronic inflammation, and prolonged exposure to carcinogenic stimuli, creating an environment conducive to gastric carcinogenesis. In this detailed review of the molecular and immune system processes involved, we have uncovered some of the core mechanisms that drive the development of gastric cancer caused by *H. pylori* infection. This revelation provides valuable insights that could lead to new treatment approaches and strategies to reduce the impact of this devastating disease. Moving forward, it is evident that further understanding of the immunological mechanisms underlying *H. pylori*-induced gastric carcinogenesis is crucial for the development of targeted prevention and treatment strategies. The exploration of novel immunotherapeutic approaches, such as immune checkpoint inhibitors or vaccines targeting *H. pylori* antigens, holds promise for more effective management of gastric cancer associated with *H. pylori* infection. Future research in this field should focus on further elucidating the intricate mechanisms underlying the immune response to *H. pylori* infection, identifying novel biomarkers for early detection, and developing targeted immunotherapies to mitigate the risk of GC development. Collaborative efforts between researchers, clinicians, and pharmaceutical companies will be crucial Overall, understanding the immunopathogenesis of *H. pylori*-induced GC is crucial for developing effective prevention and treatment strategies to combat this significant health burden worldwide.

## Author contributions

MS: Writing – review & editing. SS: Writing – review & editing. MB: Writing – review & editing. FS: Writing – review & editing. MM: Writing – review & editing. RS: Writing – review & editing. ET: Writing – review & editing. ZA: Writing – review & editing. MH: Investigation, Methodology, Supervision, Writing – review & editing. DE: Investigation, Methodology, Supervision, Writing – review & editing.
